# Pet animals as reservoirs for spreading methicillin-resistant *Staphylococcus aureus* to human health

**DOI:** 10.5455/javar.2023.j641

**Published:** 2023-03-31

**Authors:** Aswin Rafif Khairullah, Sri Agus Sudjarwo, Mustofa Helmi Effendi, Sancaka Cashyer Ramandinianto, Maria Aega Gelolodo, Agus Widodo, Katty Hendriana Priscilia Riwu, Dyah Ayu Kurniawati

**Affiliations:** 1 Doctoral Program in Veterinary Science, Faculty of Veterinary Medicine, Universitas Airlangga, Surabaya, Indonesia; 2 Department of Veterinary Pharmacology, Faculty of Veterinary Medicine, Universitas Airlangga, Surabaya, Indonesia; 3 Department of Veterinary Public Health, Faculty of Veterinary Medicine, Universitas Airlangga, Surabaya, Indonesia; 4 Lingkar Satwa Animal Care Clinic, Surabaya, Indonesia; 5 Department of Animal Infectious Diseases and Veterinary Public Health, Faculty of Medicine and Veterinary Medicine, Universitas Nusa Cendana, Kupang, Indonesia; 6 Indonesia Research Center for Veterinary Science, Bogor, Indonesia

**Keywords:** MRSA, pet animal, public health, zoonosis

## Abstract

Methicillin-resistant *Staphylococcus aureus* (MRSA) is a strain of pathogenic bacteria that is a major problem in the world’s health. Due to their frequent interaction with humans, pets are one of the main risk factors for the spread of MRSA. The possibility for zoonotic transmission exists since frequently kept dogs and cats are prone to contract MRSA and act as reservoirs for spreading MRSA. The mouth, nose, and perineum are the primary locations of MRSA colonization, according to the findings of MRSA identification tests conducted on pets. The types of MRSA clones identified in cats and dogs correlated with MRSA clones infecting humans living in the same geographic area. A significant risk factor for the colonization or transmission of MRSA is human-pet contact. An essential step in preventing the spread of MRSA from humans to animals and from animals to humans is to keep hands, clothing, and floor surfaces clean.

## Introduction

Methicillin-resistant* Staphylococcus aureus* (MRSA) is a strain of pathogenic bacteria that is a major problem in the world’s health [1,2]. The MRSA strain found in hospitals is known as hospital-acquired MRSA (HA-MRSA) and among the community as community-acquired MRSA (CA-MRSA) [3]. The broad antibacterial resistance profile of MRSA strains poses a major danger to every susceptible individual in the community and the hospital area; cases of MRSA infection are also a significant cause of human death [4].

Recent reports of MRSA transmission leading to skin and soft tissue infections, as well as the development of fatal pneumonia cases in individuals without risk factors, have raised serious concerns about this issue [5].

The home where people reside is one of the major risk factors for MRSA transmission due to its rising prevalence because a house is a place where everyone spends much time every day [6], and the house where they live is also a place for daily interactions between adults and children so that MRSA transmission can spread among family members [7].

Usually, each family has a pet in the house where they live, and pets have an important role in transmitting MRSA between family members [8]. The potential for MRSA transmission through pets is still unknown regarding risk factors for transmission between species, animal populations, or breeds with an increased risk of pets as MRSA reservoirs [9].

Because MRSA may spread from humans to animals, people who keep pets at home or work in veterinary hospitals are particularly vulnerable to colonization or infection [10]. Particularly in recent years, reports of cases of MRSA infection caused by pets have increased substantially [11].

In this review, we extensively describe MRSA, the molecular typing, the epidemiology, the transmission from animals to humans, risk factors, public health concern related to the transmission, and control of MRSA in pets.

## MRSA in Pet Animals

*Staphylococcus aureus *is a widespread bacterium found in humans and animals [12], in *S. aureus,* the gene activity that encodes penicillin-binding proteins causes a resistance reaction to beta-lactam antibiotics we know as MRSA [13], additionally resistant to non-beta-lactam medicines are MRSA strains. At first, MRSA was thought to cause nosocomial infections [14]. Nonetheless, MRSA colonization and infection have recently been found in a lot of people and animals who are not in clinical settings [15]. 

Since cases of MRSA infection are more widely known in humans in the community, it is not realized that transmission of MRSA from pets may occur due to frequent direct contact between humans and pets [16]. Human and animal health will be significantly affected by the MRSA outbreak in pets [17]. The possibility for zoonotic transmission exists since frequently kept dogs and cats are prone to contract MRSA and act as reservoirs for the spread of MRSA [18,19]. Numerous reports in recent years have indicated that pets contribute to the spread of MRSA in homes [7,20,21].

The clinical detection of MRSA colonization in pets shows that most infected pets have no issues, although opportunistic infections can start to appear [22]. However, opportunistic infections in other sections of the body are possible, and the sex and age of pets can also play a role in these infections. Surgical site infections, infections, wounds, otitis, pyoderma, and urinary tract infections have all been recorded [23]. The use of antibiotics, particularly fluoroquinolones, appears to be a risk factor for MRSA infection compared to methicillin-susceptible *S. aureus* (MSSA) infection in dogs and cats as researchers begin to look at the risk factors for MRSA transmission [24]. Compared to infections brought on by other pathogenic microbes, the effects of MRSA infection in dogs and cats are still not well understood [25]. In one study, there were no significant differences in survival rates in MRSA or MSSA-infected dogs and cats [26], although most MRSA-infected pets had otitis or pyoderma. These cases are usually far from fatal [27]. More research is required to determine how invasive MRSA infection would progress compared to MSSA infection or other infections [28].

Some healthy dogs and cats have also tested positive for MRSA [29]. Previous research has shown that healthy cats have a prevalence rate of 0%–4% for MRSA colonization [19]. It is also uncommonly understood how dominant infections colonize dogs and cats [30]. Numerous investigations have used isolates from nasal, rectal, or perineal swabs as well as swabs from other body regions [4]. The results of these variables showed that several pets were detected as positive for MRSA [31]. The causes of MRSA colonization in neighborhood pets have not been well-researched [32]. However, one long-term study compared the colonization of MRSA in dogs visiting hospitals to dogs visiting other places besides hospitals [33], then contrasted the finding of MRSA colonization in dogs exposed to hospitals and dogs that came into touch with kids as a major risk of MRSA colonization [34]. Then, among the dogs who visited the hospital, they were let to lick the patient and be fed by the person who posed a danger of spreading MRSA, which then concluded several conclusions about the various types of MRSA transmission [35].

The findings of multiple earlier investigations back up the idea that MRSA in people causes MRSA to colonize household animals [20]. In these circumstances, MRSA strains found in pets tend to predominate in humans [36]. Humans may eventually become a source of MRSA transmission in many countries if only a few have pets [37].

There is little investigation into the dynamics of MRSA colonization in pets. However, there is proof that MRSA colonization in canines and felines is just temporary [9], likely because *S. aureus* is not the prevalent commensal in canines and felines by nature [38]. This is a very important aspect because if MRSA colonization in dogs and cats is temporary, then active efforts to decolonize MRSA in dogs and cats feel unnecessary [39]. At the same time, MRSA colonization will greatly affect the health conditions of animals and humans [40].

In a UK investigation, the genomes of 46 MRSA multilocus sequence type (ST) 22 MRSA isolates from cats and dogs were sequenced and compared with related human population-wide isolates [41]. According to the phylogenomic analysis, MRSA was interspersed throughout all domesticated isolates and associated with human isolates from England [42].

In a recent study, risk factors and the prevalence of MRSA transmission from pets living in homes with an MRSA-infected family member were assessed [43]. A total of 99 pets (52 cats and 47 dogs) from 66 households where 1 family member was infected with MRSA isolates from humans and pets. After screening using a swab protocol, 11 pets (11.5%) representing 9 households (13.6%) showed positive results for MRSA; in 9 households that were confirmed positive for MRSA, 6 of them were family descendants and genetically matched animal sources [44]. Infection in humans by MRSA is significantly associated with pets [45]. However, people are not always considered MRSA reservoirs regarding the source of MRSA transmission to pets [46]. On the other hand, a rapid and precise swab examination is likely to result in a positive MRSA culture [47]. Therefore, it is important to investigate strategies to stop the spread of MRSA to humans and pets [31].

## Molecular Typing of MRSA in Pet Animals

Understanding the genetic evolution and adaption of these strains in various hosts has sparked a lot of interest in identifying MRSA in animals [48]. Molecular analysis shows that bacteria always evolve and adapt to environmental conditions, respond to selective pressure on the host body, compete with normal flora microorganisms in the host body, and use antibiotics that vary among host species [49]. Discussions on the adaptation of MRSA-infected host species and animal care facilities are very important for establishing policies for implementing control practices, not only in hospitals and animal shelters but also in residential homes [50]. Host adaptability has been demonstrated in MRSA CC133 and MRSA CC398, which includes clones in animals carrying the *vwb* gene and clones in humans having immune resistance cluster genes, including *scn*, *chp*, and *sak* transmitted by the Sa3 virus [51]. Human clones isolated were obtained from humans without a history of contact with pets. At the same time, family members and veterinarians may contract MRSA linked to pet clones. MRSA transmission risks are not limited to dogs and cats; they include exotic creatures kept as indoor pets [52]. A pet is owned by about 62% of people in the United States [53]. Most are dogs, cats, reptiles, birds, or other small animals [53]. On the other hand, animals in zoos and fish in aquariums can also be risk factors for MRSA transmission because some animal species are closely related to humans [54].

The nose, perineum, and mouth are the primary locations of MRSA colonization, according to the findings of MRSA identification tests conducted on pets [55]. Misic et al. [56] have investigated microorganisms from the body parts of pets where MRSA colonization is most common and the direct contact of pets with humans. MRSA in cats is not associated with other microbial flora communities, whereas MRSA in dogs is associated with other microbial flora [19]. On the other hand, pets can transmit microorganism flora to family members in their homes and not to other pets outside their homes [57]. These results are supported by the observation that people with pets have a flora of microorganisms that is more comparable to those without pets [58].

Due to close contact, dogs and cats were colonized or infected with MRSA by humans, according to molecular typing [59]. Colonial complexes (CCs) of MRSA in dogs and cats include MRSA CC22, CC8, CC239, CC5, and CC59 [59], while in exotic animals, several MRSA colonic complexes have been detected, including MRSA CC8 and CC22 [60].

Healthy pets still had MRSA found in 0.66% of dogs and 0.46 cats, according to a Greater London study on healthy dogs and cats still receiving care in a veterinary institution. In contrast, there was a higher prevalence rate in animals still treated in veterinary hospitals, namely in dogs at 3.23% and cats at 2.16% [61]. The clonal analysis showed that in cats, there were CC22 and CC30 clones, while in cats, there were CC22 and CC8 clones [62]. Pets carry MRSA clones, whereas type sequence characterization (ST) in pets still requires further epidemiological studies [63].

Healthy pets were screened by Larsen et al. [46] to look for MRSA colonization. In contrast, family members were split into three categories: those who worked in veterinary medicine, those who were veterinarians, and those who had no connection to the medical system. The findings revealed no discernible differences in the three human populations’ MRSA colonization rates, but the prevalence rate of MRSA colonization in pets was 3.41% [64]. Only four pairs of pets had pulsed-field gel electrophoresis (PFGE). Nonetheless, it is still unclear if the relevant strain originated from humans or the animals themselves [65].

Molecular analysis of MRSA in pets and humans in Portugal indicated that MRSA clones CC22 and CC5 detected in pets share similarities with HA-MRSA present in humans and the CA-MRSA lineage carrying toxin genes, including Panton-Valentine Leukocidin (PVL) [66]. Then another analysis showed that one MRSA CC5 clone had similarities with *S. aureus* resistant to vancomycin [67]. The findings of this study raise questions about the possibility that pets could serve as reservoirs for the spread of *S. aureus, *which is vancomycin-resistant and virulent MRSA [68].

A study of dog and cat isolates done in Germany between 2010 and 2012 revealed a greater incidence of MRSA. *Staphylococcus aureus* was identified at a prevalence rate of 5.8% in dogs and 12.2% in cats [69]. Then from the *S. aureus* isolates, MRSA identification was carried out, the results of the examination showed that the prevalence rate of MRSA identification in dogs was 62.7% and in cats, it was 46.4%; the epidemiological prevalence was 3.6% in dogs and 5.7% in cats [24]. Due to the presence of MRSA clones in humans, specifically MRSA CC22 and CC5, the results of the MRSA genotyping analysis demonstrated that humans were the source of infection for dogs and cats [70].

A comprehensive study conducted in Austria on MRSA-infected cats, dogs, and rabbits showed that of all species, ST398-SCCmec type IVa was found, while the other three strains had enterotoxin genes [71].

## Epidemiology of MRSA in Pet Animals

MRSA was initially discovered in the milk of dairy cows with mastitis [72,73]. Since then, numerous other domestic species have tested positive for MRSA, such as dogs [42,74], cats [29], horses [75,76], sheep [77,78], pigs [79,80], and chickens [81,82] increasing reports of MRSA colonization in animals as a result.

In a recent investigation, all *S. aureus* from 65 animals attending a veterinary clinic revealed that 14% of animals had MRSA infections, with dogs and cats being the most common carriers [21]. MRSA is a serious health issue, even if the rise in MRSA case reports may be a result of people being more aware of the need to test for the infection in dogs. It requires proper care [83]. Table 1 summarizes some of the incidences of MRSA infection in pets. Since MRSA was initially isolated in pets, as seen in Table 1, other incidences of MRSA infection have been documented.

MRSA infections in animals were sporadic in the late 1990s and were mostly brought on by close contact between animals and people [8]. The kinds of MRSA clones found in cats and dogs were similar to those found in humans living in the same region [42]. For instance, one strain of HA-MRSA (ST239-III) was reported to have been found in domestic dogs that were MRSA-infected in Australia [104]. Similarly, MRSA strains from canines found in Portugal and the UK are linked to the region’s predominate healthcare strain [epidemic MRSA (EMRSA)-15, ST22-IV] [108,109], similar to this, 50% of MRSA infections in pets being treated at veterinary clinics in midwestern and northeastern America were the most prevalent HA-MRSA strain in hospitals in America (USA100, ST5-II) [110]. Recently, pets in Germany, England, and France have tested positive for MRSA ST398 [105,106].

Due to cats becoming infected with MRSA, the first case of MRSA infection in humans believed to be cat-related occurred in patients and employees at a geriatric nursing home in the UK in 1988 [84]. There is a chance that direct contact between animals and people could increase the danger of MRSA spreading [31]. In particular, those with pets are more likely to contract MRSA than people without pets [20], showing that pets serve as a source of MRSA transmission [21].

In addition, several studies have revealed that MRSA in pets is mostly derived from MRSA in humans [16,44,128]. The risk of MRSA colonization is correlated with close interaction with humans and pets [20].

## Transmission of MRSA from Pet Animals to Humans and from Humans to Pet Animals

According to the findings of epidemiological research carried out in many countries, harmful bacteria resistant to antibiotics can be transmitted between humans and pets [129]. Pet isolates in the UK, where the spread of MRSA was examined among animals, medical personnel, and the environment, included PFGE that was the same as that of disseminated EMRSA-15, a clone of HA-MRSA in the UK [98]. The identification of similar HA-MRSA clones has also been found in pets and hospital staff in a study conducted in several veterinary clinics in Ireland [97]. In Korea, HA-MRSA ST5-II clones have also been discovered in hospitalized dogs and people, demonstrating that MRSA transmission can happen between pets and people and between people and animals [130]. The same clone was identified among humans, cats, and dogs, related to USA 100, which is an HA-MRSA clone and can cause community infection in Canada [103].

In addition to clones, transmission between toxigenic strains can also occur. Studies on the identification of enterotoxigenic *S. aureus* isolates in humans and animals have shown this [115,131], and this allows for the spread of CC with specific toxigenic profiles [110]. In a study conducted in Greece, 9 strains were identified from 5 humans and 29 pets, among these 9 MRSA strains, there were 5 detected PVL-positive strains and had ST80, a widespread CA-MRSA clone in Greece [128].

Variances in MRSA epidemiology, length of close contact, sample period, and established technique for looking for MRSA reservoirs may be explained by differences in MRSA infection or colonization between pets and people [132]. Most MRSA isolates detected in pets have MRSA clones that can cause human infection, and the transmission of MRSA also depends on the epidemiology of a particular geographic area [1]. Veterinarians can also contract MRSA from animal patients they care [94]. Applying infection prevention techniques and policies to the use of antibiotics in veterinary medicine will become necessary based on epidemiological results and molecular analyses of the transmission of MRSA [133].

## Risk Factors of MRSA Infection in Pet Animals

Veterinarians at veterinary clinics in Japan looked at risk variables for MRSA infection or colonization [134]. Veterinarians have a 22.9% MRSA prevalence rate, and most male veterinarians have risk factors for MRSA colonization [135]. Despite reports of human contact with animals, there is no statistically supported link between this factor and MRSA transmission in people [136]. Given that Malaysia is one of the nations with a high prevalence of MRSA, a molecular analysis of MRSA strains carried out in Malaysia revealed that there is a possibility for MRSA transmission among dogs, veterinarians, and the environment [137].

Even though there have been numerous infections with MRSA that share the same clones as MRSA in humans, in a study conducted in Spain on six farms with pigs that were detected positive for MRSA ST398, the results of cluster and similarity analysis proved that the strain of MRSA ST398 in pigs came from pets roaming around the farm, humans in contact with pigs, rats, and from the environment [138]. Pets and other small animals near farms, breeders, and the environment can be susceptible to MRSA transmission [139].

**Table 1.** Several reports of MRSA infection in pets.

Year	Chronology	Location	References
1988	Cats suspected as reservoir of MRSA transmission in geriatric wards	UK	[84]
1989	Isolation of coagulase-positive *S. aureus* from orthopedic swab implants in dogs (12% of coagulase-positive *S. aureus* isolates were resistant to oxacillin)	USA	[85]
1994	MRSA colonization of the noses of two nurses suspected of being infected by a pet dog	UK	[86]
1998	Detection of MRSA from cat skin scrapings that are still in good health	Brazil	[87]
1999	Detection of MRSA in 12 dogs with various clinical conditions admitted to a veterinary hospital	South Korea	[88]
	MRSA infections that occurred in 11 dogs as a result of surgery, recurrent pyoderma, or traumatic injuries	USA	[89]
	Isolation of MRSA from skin lesions and scars in dogs	USA	[90]
2003	Discovery of identical PFGE-type MRSA isolated from domesticated dogs is associated with recurrent MRSA infection	USA	[91]
2004	MRSA isolation from scars in two dogs	Germany	[92]
	A total of 95 MRSA isolates were found in cats, dogs, and rabbits. Most of the MRSA isolates were obtained from postoperative skin infections and skin wound infections	UK	[93]
	A total of 12 MRSA isolates were detected in cats and dogs	UK	[94]
	Environmental contamination that is the source of MRSA transmission in veterinarians teaching hospitals	Canada	[95]
2005	Isolation of MRSA from a non-healing abscess in a cat	USA	[96]
	MRSA isolates from cats, dogs, and rabbits had identical PFGE to human MRSA strains	Irish	[97]
	Isolate MRSA from dogs, staff, and veterinary hospital environments	UK	[98]
	The prevalence rate of MRSA colonization in dogs is 1% in referral veterinary hospitals	Canada	[99]
	Isolation of MRSA from staff and dogs in a veterinary hospital identical to PFGE analysis for MRSA strains in humans	UK	[100]
2006	Prevalence of MRSA colonization rates in dogs which by 0.6% increased to 8%	Ireland	[34]
	Isolation of MRSA from postoperative infection scars in five dogs and veterinarians	Ireland	[101]
	MRSA isolates from cats and dogs were similar to MRSA isolates from hospitals in the human population	Germany	[102]
	Transmission of MRSA from humans to animals and from animals to humans after investigation	Canada	[103]
	The *mec*A gene in MRSA isolates from cats and dogs is identical to the *mec*A gene found in humans	Australia	[104]
2007	The presence of nuc and *mec*A was confirmed for MRSA were identified in cats and dogs	UK	[105]
	MRSA of clonal lineage ST398 that exhibits related spa types and contains SCC*mec* elements of types IVa or V has been isolated from colonized and infected companion animals	Germany and Austria	[106]
	MRSA carriage was identified in 10/129 dogs (7.8%) dogs and all isolates were of the same lineage as the one isolated from the infected dog	UK	[107]
2008	*spa* typing and DNA microarray analysis of resistance and virulence genes was carried out on all MRSA of dogs	UK	[108]
	Four different spa types were identified among our MRSA isolates (t032, t432, t747, and t4726), with t032 as the most frequently detected from nasal swabs of 54 healthy dogs	Portugal	[109]
	MRSA was present in clinical samples from 12 of 487 (2.5%) dogs and 6 of 48 (12.5%) cats in veterinary clinics	USA	[110]
2009	The prevalence of concurrent MRSA colonization in 1 of 10 (10%) cats and 2 of 24 (8.3%) dogs	USA and Canada	[111]
	Dogs have been found to be colonized by the livestock-associated LA-MRSA clone characteristic of food animals and identified as ST398	UK	[105]
	Nine MR isolates (27%) carrying the *mec*A gene were detected (eight MRSP and one MRSA) from diseased pets	Spain	[112]
2010	High MRSA rates were identified at 62.7% and 46.4% in *S. aureus* of canine and feline origin	Germany	[69]
	The companion animals of 49 MRSA-infected outpatients (cases) were screened for MRSA carriage, and their bacterial isolates were compared with those of the infected patients using PFGE	USA	[31]
	Fifty percent of the dogs with pyoderma showed MRSA isolates that were resistant to almost all the antimicrobials used in the present study	India	[113]
2011	MRSA was isolated from dogs (1%), and most of MRSA ST398 carried SCC*mec* type V	Thailand	[114]
	Three MRSA isolates have been recovered from 2 dogs out of 70 (2.9%) and none of the examined cats yielded MRSA	Egypt	[115]
	A case-control study was conducted, 106 MRSA-infected animal patients (dogs and cats) as cases	Germany	[24]
2012	MRSA was isolated from one cat and one dog, isolates from the two animals were genotype USA300 (community-associated strains) and were indistinguishable by PFGE from the case patient’s initial infection isolate	USA	[116]
2013	The study reports the first case of MRSA strains in dogs in Kenya, which were associated with mobile genetic elements(SCC*mec*)	Kenya	[117]
	The estimated true MRSA subclinical colonization prevalence was 1.9% for cats and 1.4% for dogs	New Zealand	[118]
2014	Twenty-two MRSA strains isolated from various infected locations in domestic cats and dogs were analyzed for their genotype, genetic fingerprint, virulence, and antibiotic resistance profile	Switzerland	
	51.51% of the canine and 84.62% of feline MRSA isolates indicated resistance to four out of five antibiotics tested	Malaysia	[119]
	Four samples (13.8%) from dogs were MRSA-positive, but samples from cats were MRSA-negative.	Bangladesh	[120]
2015	Of 132 pets, 14% were colonized with MRSA, and pets whose primary caretaker was MRSA-colonized were more likely to be MRSA-colonized	USA	[39]
2016	Ten of a total of 129 dogs at a rescue facility were found positive for MRSA at mucosal and skin carriage sites, whereas all 16 companions sharing a kennel with one of the carriers were negative	USA	[26]
2017	4 (4.3%) MRSA isolates were found in 93 samples (nasal swabs and wound swabs) of dogs and cats at veterinary hospitals and animal markets	Bangladesh	[121]
2018	A total of 20 MRSA isolates of 134 *S. aureus* isolated from canine and feline clinical samples were CC398, consisting of ST 398 (18 isolates), ST5926 (1 isolate), and ST6563 (1 isolate) by multilocus sequence typing	Thailand	[122]
2019	33.66% *mec*A genes harboring *S. aureus* strains were isolated from all sources (33.33% from pets, 46.0% from surrounding, and 28.0% from immediate contact individuals)	Pakistan	[123]
	*Staphylococcus aureus* were detected in 11 (15.4%) dog samples of which 5 were MRSA	Portugal	[16]
2020	MRSA confirmation by oxacillin-resistant screen agar base (ORSAB) was 25 (29.41%) from nasal swabs of dogs in Surabaya. The molecular identification of *mec*A gene by polymerase chain reaction showed that 5 (5.88%)	Indonesia	[18]
	Fourteen isolates from nasal swabs of dogs were screened for MRSA by culture on ORSAB	Indonesia	[124]
	MRSA detected from dog swabs showed a rate of 44.4% and cat swabs revealed 27.3%	Saudi Arabia	[125]
2021	A total of 730 samples from skin infections of dogs and cats that tested positive for bacterial growth, 27 (3.7%) were *S. aureus*. The isolate tested for oxacillin *n* = 4, it was MRSA	Portugal	[126]
2022	The findings of the identification and isolation process revealed 18 (12%) *S. aureus* isolates from nasal swabs of cats	Indonesia	[127]

An important risk factor for MRSA transmission can also be through pets other than dogs and cats [136]. MRSA infections have been identified in parrots, hamsters, iguanas, turtles, and small ruminants [140]. The risk factors for MRSA transmission in these animals are still relatively small and are only occasionally discovered, even though they do not frequently interact with humans. However, reports of MRSA infections in parrots, bats, rabbits, and turtles have been made in a small veterinary clinic in Berlin [141]. The ST22-SCC*mec*IV MRSA has the same lineage as human MRSA [142].

The association between MRSA isolates from pets and humans was similar to MRSA among household members [20]. About 50%–67% of MRSA in households have a similar MRSA [143]. Although it has been found that the transmission of MRSA strains is associated with humans and pets, it can be from humans to pets or pets to humans; therefore, humans and pets are the main risk factors for MRSA transmission [31]. On the other hand, the surroundings of the home can potentially provide a risk for MRSA transmission [144].

## Public Health Concern

MRSA contamination of humans, animals, and food is now a significant public health issue [145]. Most of these worries stem from the possibility that animals could serve as MRSA reservoirs and spread the disease to people [146].

There have been numerous reports of MRSA colonization in humans who have come into touch with MRSA-infected small animals [8]. However, some have led to clinical zoonotic infections. The majority of studies have shown MRSA colonization [46]. Pets have served as reservoirs for the domestic transmission of MRSA to humans [10]. Still, this direction of MRSA transmission remains unclear, even if the same strain of MRSA is identified in both animals and humans [147]. The results of molecular typing analysis cannot be used to pinpoint the source of MRSA zoonotic infection, especially concerning pets, because they frequently have the dominant type of MRSA in people [16]. People who work in animal hospitals have also been diagnosed with zoonotic infections [148].

The rate of MRSA colonization and the study population among persons in the general population should be considered in all studies about MRSA in pets [44], in general, it has been documented that veterinary employees are colonized with MRSA at comparatively high rates [132]. It has not yet been determined with certainty that this level of MRSA colonization is acquired by MRSA from pets [21]. Data from molecular typing studies show that veterinarians have infected small animals with a preponderance of related MRSA clones [122].

Numerous studies have assessed risk factors, and one of the most important ones for the spread or colonization of MRSA is contact with pets [43]. The statistically significant preventive impact of routine hand washing following contact with instances of contagious animal diseases and between households was the most noteworthy finding [149], showing the value of common infection prevention techniques like hand washing for MRSA control [150].

## Control of MRSA in Pet Animals

The Centers for Disease Control and Prevention has issued several documents on the management of MRSA in people [151], and many of the MRSA control principles are also applicable to pets [44]. However, caution must be taken when separating recommendations for MRSA control in humans from those for controlling the disease in pets, as there may be considerable variations in disease epidemiology [31]. As of yet, no controlled studies have been conducted to offer information on important issues, including prevalence, infection, the permanence of MRSA colonization in pets, the efficacy of decolonization treatments in pets, and the simplicity of MRSA transmission between people and pets [19].

Previous studies that looked at risk factors for MRSA transmission found that past antibiotic use, rates of previous colonization, and knowledge of prior MRSA infections all posed a threat to the spread or colonization of the bacteria [152]. Additionally, while it is most likely that the veterinary professionals who work with small animals would be the main source of MRSA infection, the environment in veterinary hospitals may also be a source of MRSA transmission due to extensive contamination [10].

Hand washing is crucial in preventing the spread of MRSA from humans to animals and from animals to humans, just like in human medicine [153]. Wash hands after contact with pets and disinfect equipment, floor surfaces, and table surfaces after handling pet patients [154]. Make sure hand sanitizer is available at home, in animal health care rooms, and in pet cages [155].

Other regular precautions against the transmission of MRSA include routinely hand-washing clothing and donning gloves when dealing with patients who have small animals, especially pets who are experiencing cases of infectious disease, aprons are expected to be disposable only, and always wearing a mask to protect yourself from contaminated air or pet body fluids [156]. The use of eye protection is also recommended if splashes or aerosols from pets are expected [157]. Other aseptic techniques include sterilizing surgical equipment before and after surgery [158]. The flooring of veterinary clinics and residences must also be carefully mopped [159].

In addition, there is a need for close collaboration between human doctors and veterinarians to identify the types of MRSA that may be found in humans and pets so that effective control measures can be taken in veterinary practice [136,160,161].

## Conclusion

This review concludes that pets and humans can be sources of MRSA transmission. The worldwide finding is that cats and dogs, and small exotic animals kept by humans, can become infected or colonized with HA-MRSA or CA-MRSA clones. It is necessary to put control measures in place to stop the spread of MRSA from animals to people. Additional research and molecular analysis of MRSA strains in pets and humans are still required because the risk factors for MRSA transmission from humans to pets and from pets to humans have not yet been fully understood.

## List of abbreviations

CA-MRSA: Community-acquired methicillin-resistant *Staphylococcus aureus*; CC: Colonial complex; EMRSA: Epidemic methicillin-resistant *Staphylococcus aureus*; HA-MRSA: Hospital-acquired methicillin-resistant *Staphylococcus aureus*; MRSA: Methicillin-resistant *Staphylococcus aureus*; PFGE: Pulsed-field gel electrophoresis; SCC*mec*: Staphylococcal cassette chromosome *mec*; ST: Sequence type; UK: United Kingdom; USA: United States of America.
